# New Appliances and Things Medical

**Published:** 1907-05-18

**Authors:** 


### NEW APPLIANCES AND THINGS MEDICAL.

### CALOX DENTIFRICE.

(McKeeson and Robbins, New York. British Agent: A. C. Wootton, 14 Trinity Square, London, E.C.)

This is an amorphous white powder; it contains an appreciable percentage of calcium dioxide, from which hydrogen peroxide is easily liberated. Used on a soft brush with ordinary lukewarm water, nascent $H_2O_2$, one of the most powerful deodorants and antiseptics we possess, is produced. Its use should keep the teeth white and prevent decomposition in the oral cavity. The powder, which is put up in specially made tins, possesses a slight but pleasant aromatic odour, and we have found in it no ingredient which is likely to harm the teeth or mucous membrane.

### WRIGHT'S COAL-TAR INHALER AND VAPORISER.

This is a simple block-tin vaporiser, fitted with an absorbent block, which is heated by a night-light. By means of it not only the coal-tar preparations supplied, but creosote, formaldehyde, and other volatile antiseptics and deodorants may be vaporised. It is cheap, portable, and light, and its simplicity is such that anyone can use it. In cases where vapour treatment is indicated it should prove of great service. The makers are Messrs. Wright, Layman and Umney, Limited, 48 Southwark Street, London, S.E.

